# Quantum-dot microlasers based on whispering gallery mode resonators

**DOI:** 10.1038/s41377-021-00525-6

**Published:** 2021-04-15

**Authors:** A. E. Zhukov, N. V. Kryzhanovskaya, E. I. Moiseev, M. V. Maximov

**Affiliations:** 1 Laboratory of quantum optoelectronics, National Research University Higher School of Economics, Kantemirovskaya 3A, St. Petersburg, 194100 Russia; 2 Nanophotonics laboratory, Alferov University, Khlopina 8/3, St. Petersburg, 194021 Russia

**Keywords:** Photonic devices, Diode lasers

## Abstract

The subject of this paper is microlasers with the emission spectra determined by the whispering gallery modes. Owing to the total internal reflection of light on the sidewalls, a high Q-factor is achieved until the diameter is comparable to the wavelength. The light emission predominantly occurs in the plane of the structure, which facilitates the microlaser integration with other elements. We focus on microdisk lasers with various types of the In(Ga)As quantum dots (QDs). Deep localization of charge carriers in spatially separated regions suppresses the lateral diffusion and makes it possible to overcome the undesirable effect of non-radiative recombination in deep mesas. Thus, using conventional epitaxial structures and relatively simple post-growth processing methods, it is possible to realize small microlasers capable of operating without temperature stabilization at elevated temperatures. The low sensitivity of QDs to epitaxial and manufacturing defects allows fabricating microlasers using III–V heterostructures grown on silicon.

## Abstract (web summary)

The use of epitaxial In (Ga)As quantum dots and relatively simple post growth methods make it possible to realize microdisk lasers capable of operating without temperature stabilization at elevated temperatures.

## Graphical abstract

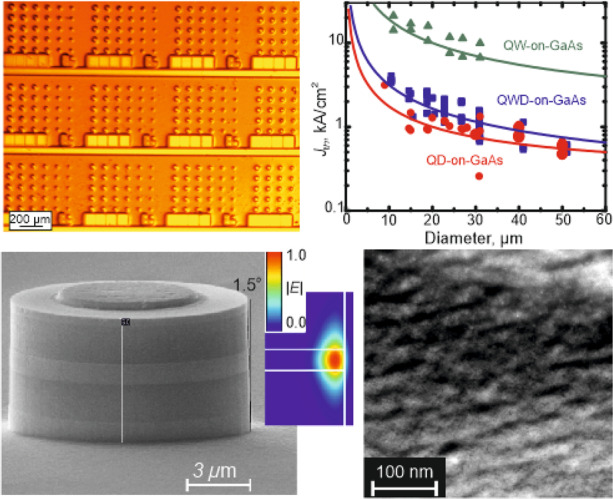

## Introduction

In recent years, the use of optical communication lines for data exchange between a processor and memory has been actively discussed and already partially implemented^1^. In the ultimate case, optical signals can probably be used to interconnect photonic and microelectronic elements in a single chip. The laser miniaturization, required for a denser device arrangement, also contributes to lowering the threshold current, facilitates single-frequency lasing, and also promotes an increase in the direct modulation bandwidth^2^. Soon after their invention^3^, the ability to reduce the lateral size down to <10 µm was demonstrated in vertical cavity surface-emitting lasers (VCSELs)^4–6^. Together with the progress in epitaxy of strained quantum wells and in methods of lateral blocking of charge carriers^7^, this made it possible to achieve threshold currents <0.1 mA^8^, modulation frequencies over 10 GHz^9^ and power consumption <100 fJ/bit^10^.

The use of microlasers in the computers or cellphones of the future would be greatly facilitated by a low cost of the laser, which can be achieved in case of simplification of its design and manufacturing method. The ability to integrate light emitters with silicon-based components is also very useful. However, the VCSEL structure, which comprises distributed Bragg reflectors, layers of gradient compositions, and oxidized apertures, is quite complex. Vertical light emission is not convenient for the integration^11^, because it requires additional elements such as 45° microreflectors^12^, integrated gratings^13^, microprisms^14^, etc. All this, despite the significant success in VCSELs, motivates the search for microlaser designs that are simpler than VCSELs, have a lateral light outcoupling, and can be integrated with Si-based elements, even if such microlasers have slightly worse performance. All the above characteristics can be realized with the help of optical microcavities supporting whispering gallery modes (WGM), as lasers can be implemented using a thin layer sequence, very similar to that is commonly exploited in edge-emitting lasers. No thick distributed Bragg reflectors, layers to be oxidized, or complicated compositional profiles are required.

When the in-plane size shrinks to tens or even several micrometers, non-radiative recombination at the sidewalls can become a serious problem. The first WGM microlasers were fabricated using the InGaAsP/InP material system^15,16^, the advantage of which is an unusually low velocity of surface recombination (≤10^4^ cm/s^17^), i.e., more than an order of magnitude lower than that for (In)GaAs/(Al)GaAs QWs^18,19^. Meanwhile, InGaAsP/InP and, albeit to a lesser extent, AlGaInAs/InP materials suffer from low heterojunction band offsets^20,21^, which leads to a poor temperature stability^22^. The application of InGaAs/(Al)GaAs materials in WGM microlasers would be problematic if not for quantum dots (QDs). The reduced diffusion length and lowered velocity of surface recombination can prevent an increase in the threshold current density in small devices^23^. It has been revealed that deep etching of a QD material, unlike QWs, does not result in degradation of the laser performance^24^. This behavior is explained by a strong reduction in the diffusion length from several μm in InGaAs QWs to ~0.1 μm in InAs/GaAs QDs^25^. Moreover, a low surface recombination velocity (~5 × 10^4^ cm/s) was found in QD edge-emitting lasers^26^. A reduced sensitivity of QDs to defects also contributes to the achievement of acceptable laser characteristics even with highly defective materials, e.g., grown on a non-parent substrate. In view of the above, we focused on microdisk lasers with QD-active region.

### Epitaxial structures for microlasers

For microlaser fabrication, we used a separate confinement Al_*x*_Ga_1−*x*_As/GaAs heterostructure. The waveguide thickness and AlAs mole fraction *x* is always chosen to support the fundamental vertical mode only. For *x* = 0.35, this corresponds to ~0.6 µm. Because GaAs spacers are typically 35–50 nm thick^27,28^, this gives enough space for at least 10 planes of QDs, including those emitting ~1.3 µm. Possible vertical alignment of QDs was not taken into account when choosing the thickness of the spacer layers.

We exploited several types of epitaxial structures. The first type structures were grown on *n* + -GaAs (100) substrates by molecular-beam epitaxy (MBE) by Innolume (Dortmund, Germany). They comprise several planes of InAs/InGaAs Stranski–Krastanow (SK) QDs separated with GaAs spacers. The active region is deposited from solid elemental sources at lowered temperature (~480 °C). First, initial InAs QDs are deposited in SK growth mode and then they are overgrown with a thin InGaAs layer to tune the emission wavelength. The amount of InAs in the initial QDs is typically ~2.5…2.7 monolayers, whereas the InGaAs covering layer has a thickness of ~5 nm and the InAs mole fraction of ~15%. The emission wavelength (ground-state (GS) optical transition) is ~1.27…1.29 µm. This sort of QDs has previously allowed achieving very low-threshold current densities^29^ and high temperature stability^30^ in edge-emitting (macro) lasers, so it was very natural to test them as the active region of injection microlasers. Hereinafter, microlasers of this type are referred to as QD-on-GaAs.

**Fig. 1:**
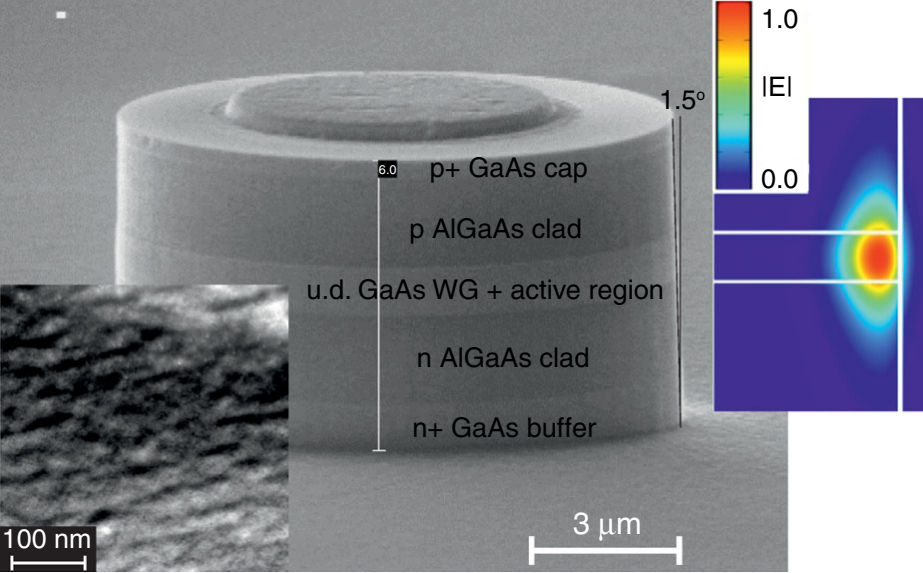
**Microdisk laser.** Main panel: SEM image of QD-on-GaAs microdisk with top contacts. Insets: plan-view TEM image of QWD array (left panel); electric field distribution (right panel)

The structures for microlasers of another type, which we called QWD-on-GaAs, are grown by metal-organic vapor phase epitaxy at the Ioffe institute (St. Petersburg, Russia) on GaAs substrates misoriented off (100) plane by 6°, which promotes transformation of InGaAs thin layers of a moderate indium composition (~40%) into a dense array of islands referred to as quantum well-dots (QWDs)^31^. Trimethylgallium, trimethylindium, trimethylaluminum, and arsine were used as precursors; temperature of the active region growth was ~500 °C. The effective thickness of the deposited InGaAs was ~2 nm. The QWD layer represents a dense array of In-rich islands with typical lateral size of ~20–30 nm and a height of ~3 nm. A characteristic plan-view TEM image is presented in the left-hand inset to Fig. 1. The islands tend to group into elongated nanowire-like objects; the surface density of the islands in the mid of 10^11^ cm^−2^, i.e., ~10 times higher than that of SK QDs. Our idea was to investigate the influence of the parameters of the active region on the performance of microlasers: QWDs possess an order of magnitude higher optical gain compared with conventional Stranski–Krastanow QDs;^32^ their spectrum is shifted to shorter wavelengths (~1.05 µm).

The majority of the experimental results discussed in the present paper were obtained for microlasers of the above-mentioned types. Still another type of heterostructures we exploited was developed at UCL (London, UK). They also contain multiply stacked arrays of MBE-grown InAs/InGaAs QDs described above. The distinguishing feature is that the III–V layers are monolithically grown on silicon substrate with additional transient layers including a short-period superlattice and dislocation filters to reduce the density of threading dislocations below 10^6^ cm^−2 ^^33,34^. The structural properties of QDs grown on Si are very similar to those grown on GaAs except density of defects. The Si (100) substrate is 4° misoriented toward [011], which facilitates single-domain growth. Broad-area lasers made of epitaxial structures of this sort have demonstrated promising threshold and reliability characteristics. Our goal was to investigate the possibility of their application to create microdisk lasers monolithically integrated with silicon (QD-on-Si). When discussing the threshold characteristics of microdisk lasers, we also present the data we obtained for a structure with triple Ga_0.7_In_0.3_N_0.02_As_0.98_ quantum wells (QW-on-GaAs) grown by nitrogen plasma MBE an *n* + -GaAs (100) substrate at TUT (Tampere, Finland). The emission wavelength is ~1.25 µm; thus, the localization energy of charge carrier in the active region is close to that in QD-on-GaAs. These microlasers provide an opportunity to study the effect of active region quantum dimensionality on the device performance. Table 1 summarizes the types of microlasers we studied and their main features.

**Table 1:** Summary of microlasers

Notation	Growth method	Active region	Quantum dimensionality	Substrate	Emission wavelength, µm
QD-on-GaAs	MBE	InAs/InGaAs	0D	GaAs(100)	~1.28
QWD-on-GaAs	MOCVD	In_0.4_Ga_0.6_As	0D	GaAs 6° off	~1.05
QD-on-Si	MBE	InAs/InGaAs	0D	Si 4^o^ off	~1.3
QW-on-GaAs	MBE	InGaAsN	2D	GaAs(100)	~1.25

### Microcavity structure

**Fig. 2:**
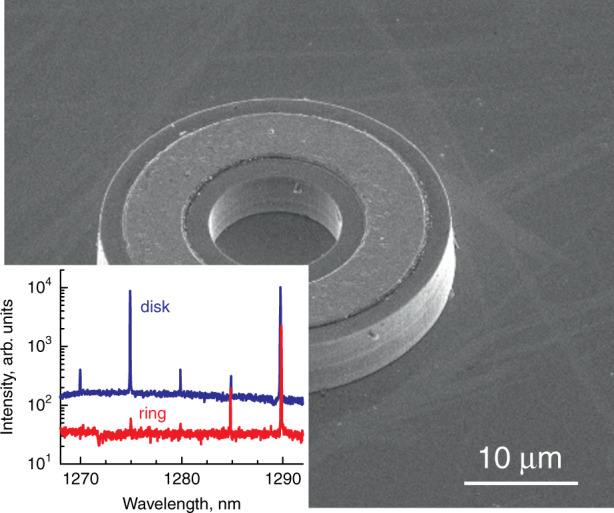
**Microring laser.** Main panel: SEM image of QD-on-GaAs microring. Inset: comparison of emission spectra of microdisk (upper curve) and microring (lower curve) made of the same structure

The epitaxial structures of all types are processed into cylindrical mesas of different diameters *D* varying from 10 to 50 µm, which are defined photolithographically. No passivation or coating is applied. A wide variety of etching techniques have been used and we now rely upon the inductively coupled BCl_3_/Ar plasma dry-etching process. The sidewall verticality within 5° is resulted, Fig. 1. Although the mode intensity falls off quickly into the depth of the cladding layer (right-hand inset to Fig. 1), we found that the etching front should go deep enough, so the mesa height is ~5 µm. Otherwise, a sharp increase in the threshold or even complete absence of lasing is resulted. We also investigated microrings (Fig. 2), as well as more complex shapes, e.g., race-tracks. Since the WGMs are concentrated at the periphery of the microcavity, we observe very little effect of the inner holes on Q-factor, inter-mode distance, mode spectral positions (inset to Fig. 2), and, to a lesser extent, on their intensities. Moreover, the removal of some of the active material leads to a decrease in the current required for the population inversion. We guess, for this reason some research groups prefer to deal with microrings rather than disks (e.g., ^35,36^). However, our opinion is not so straightforward, as an additional open surface can cause additional non-radiative recombination, and a smaller footprint increases the electrical and thermal resistances.

### Electrical contacts and resistance

**Fig. 3:**
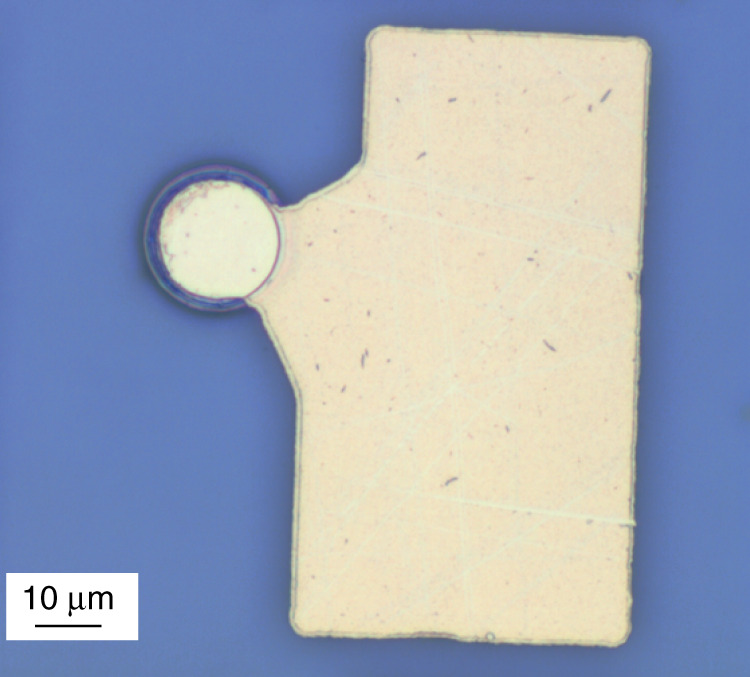
**Planarization of microlaser.** Planarized QWD-on-GaAs microdisk with contact pad placed aside

**Fig. 4:**
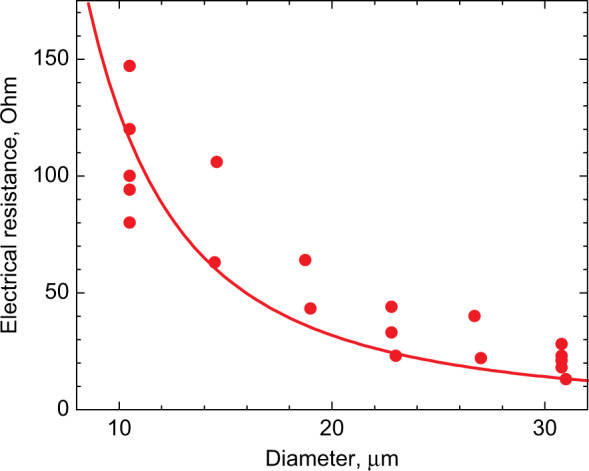
**Electrical resistance.** Series electrical resistance of QWD-on-GaAs microdisk lasers as a function of diameter; line correspond to specific resistance of 10^−4^ Ω × cm^2^

Electrical *n*- and *p*-contacts are made using AgMn/Ni/Au and AuGe/Ni/Au metallization, respectively. Top *p-*contacts have a few µm smaller diameter than the mesa itself. The mesas can be planarized, e.g., with SU-8 epoxy dielectric, and larger contact pads are made over the coating, Fig. 3. A common *n*-contact is usually placed on the bottom side of the conductive GaAs substrate. The substrate electrical resistance $1/(2D\sigma )$ is described using the model of current spreading in a semi-infinite medium with conductivity $\sigma$^37^. Unlike large-area mesas, it turns out to be small compared with the resistance of the mesa itself, which is $R_{{\mathrm{bottom}}} \approx \rho _{{\mathrm{disk}}}\pi (D/2)^2$ with the specific resistance $\rho _{{\mathrm{disk}}}$≈1 × 10^−4^ Ω × cm^2^, Fig. 4. We did not find a significant difference in the electrical characteristics of microdisk lasers on a conductive GaAs substrate made from different epitaxial structures we exploited.

**Fig. 5:**
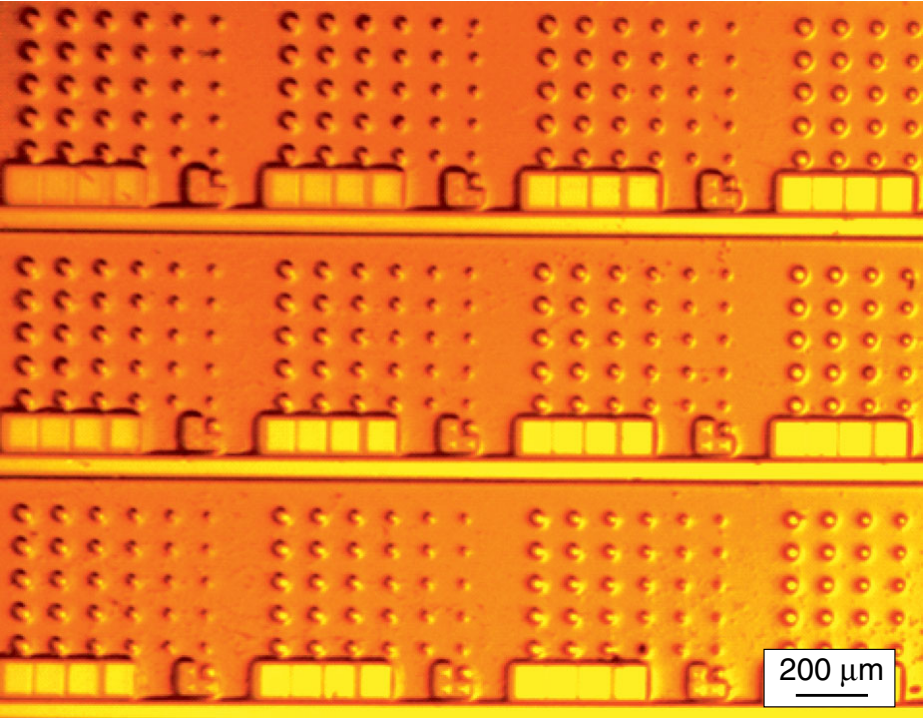
**Arrays of microdisks.** 6 × 5 arrays of QD-on-Si microdisks of different diameters (11, 15…31 μm) with gold-plated buffer layer between mesas

**Fig. 6:**
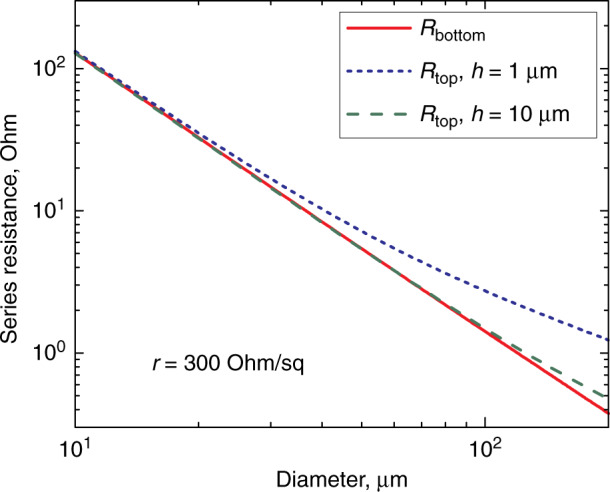
**Impact of the** ***n*-contact location.** Calculated microdisk resistance for conductive *R*_bottom_ (bottom-contact; solid line) and non-conductive *R*_top_ (top-contact; dashed and dotted lines) substrate with the conductive buffer of thickness *h*

For structures on Si, the *n*-contact covers the *n* + -GaAs buffer between the microdisks, Fig. 5. The resistance is determined by the current crowding at the contact edges^38,39^. We found^40^ it can be approximated as $R_{{\mathrm{top}}} \approx \left( {\sqrt {\rho _{{\mathrm{disk}}}r} /\pi D} \right)I_0\left( {\frac{D}{{2\sqrt {\rho _{{\mathrm{disk}}}/r} }}} \right)/I_1\left( {\frac{D}{{2\sqrt {\rho _{{\mathrm{disk}}}/r} }}} \right)$, *r* being sheet resistance of the buffer, $I_m$—modified Bessel function of the first kind of the *m*-th order. If the disk diameter is large, $R_{{\mathrm{top}}} \approx \sqrt {\rho _{{\mathrm{disk}}}r} /(\pi D)$, which noticeably exceeds $R_{{\mathrm{bottom}}}$ for typical thicknesses of the buffer, Fig. 6. For small diameters ($D \lesssim$ 30 µm), the asymptotic behavior of the Bessel functions gives $R_{{\mathrm{top}}} \approx \rho _{{\mathrm{disk}}}/\pi (D/2)^2$, i.e., top location of the *n*-contact can be used in this case without compromising the resistance.

### Spectral characteristics and optical loss in QD WGM microdisks

**Fig. 7:**
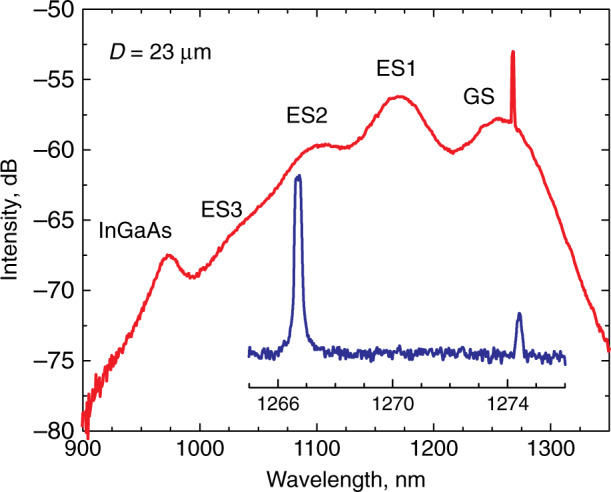
**Emission spectra.** Main panel: whole emission spectrum of QD-on-GaAs microdisk. Inset: spectrum taken with a higher resolution near GS maximum

**Fig. 8:**
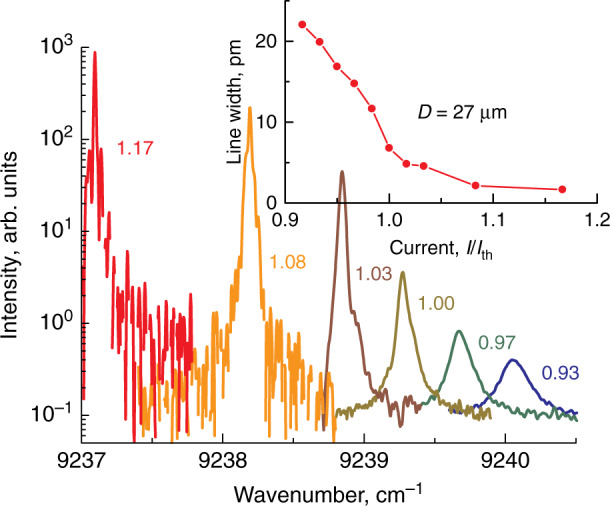
**High-resolution emission spectra.** Main panel: high-resolution spectra of QWD-on-GaAs microdisk; numbers indicate the injection current normalized to the threshold current *(I*/*I*_th_). Inset: linewidth (FWHM) against normalized injection current

**Fig. 9:**
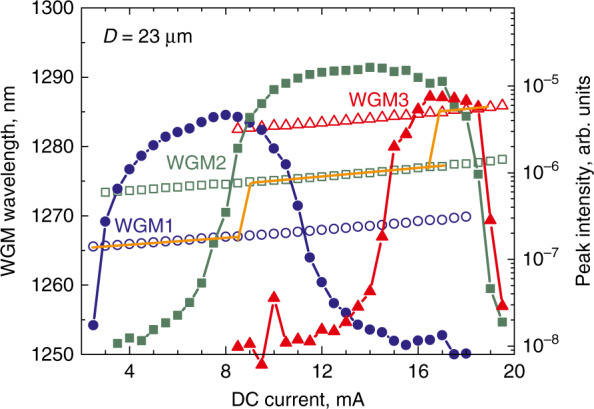
**Mode wavelength and intensity.** Spectral position (open symbols) and line intensity (solid symbols) of three brightest WGMs of QD-on-GaAs microdisk as a function of injection current

The emission wavelengths of microlasers are controlled by the wavelengths of the GS optical transition of the active region, which in turn is set by their specific features, such as quantum dimensionality, chemical composition, growth regime, etc., as summarized in Table 1. WGMs manifest themselves as sharp lines located near the maximum of the inhomogeneously broadened QD GS optical transition, Fig. 7. Usually, the spectra exhibit from one to four modes simultaneously with a separation between them of ~5…10 nm being inversely proportional to *D*. The mode intensity starts to grow sharply upon reaching the threshold current, Fig. 8, which evidences the onset of lasing. Similar behavior is also observed, when QWD active region is used. The WGM lasing is further confirmed by narrowing the mode linewidth (inset to Fig. 8), as expected from the Schawlow-Townes laser linewidth equation^41^. At high injections, a gradual ignition of the adjacent longer-wavelength WGM is observed, Fig. 9, whereas the initial lasing mode declines. Nevertheless, at certain currents, the side mode suppression ratio is as high as 25…35 dB, i.e., the laser is quasi-singlemode. Still higher currents can ignite the next mode, so the spectral position of the brightest line jumps from one WGM to another. This phenomenon appears to be similar to the two-state lasing in quantum-dot edge-emitting lasers^42,43^, but its nature is quite different. Different explanations for the origin of two-state lasing in QDs^44–47^ always involve a shorter-wavelength (excited-state) optical transition. In contrast, the lasing wavelength of the microlasers is red-shifted remaining within the GS band, and thus can be caused by an increase in temperature and a resulting shift of the gain spectrum.

**Fig. 10:**
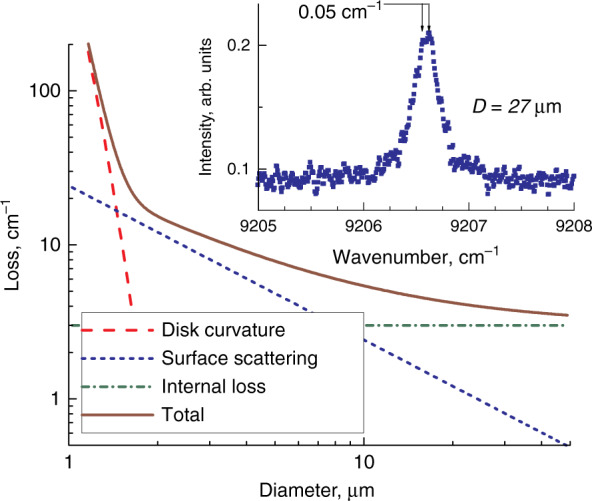
**Optical loss.** Main panel: calculated optical loss and its components as a function of microdisk diameter. Inset: high-resolution below-threshold spectrum of QWD-on-GaAs microdisk

Q-factor of a WGM in a perfect dielectric cylinder is limited by the light emission through the side surface^48,49^, and the loss sharply increases as soon as the diameter becomes comparable to the wavelength, Fig. 10. Sidewall roughness causes additional loss, which depends on the effective volume of the light scatterers^50^. It can be estimated from the splitting of a single WGM line into a doublet owing to the lift of the degeneracy of waves propagating clockwise and counter-clockwise^51,52^, inset to Fig. 10. Suggesting that the surface roughness amplitude is independent of *D*, the scattering induced loss $\propto D$ and becomes below 2 cm^−1^ for diameters exceeding 10 µm. The free carrier absorption and some other mechanisms known as internal loss in properly optimized QD- and QWD-based edge-emitting lasers can be $\lesssim$1…2 cm^−1 ^^29,53,54^, so the total optical loss in QD-on-GaAs microdisks of typical size is expected to be about few cm^−1^.

### Threshold current density

**Fig. 11:**
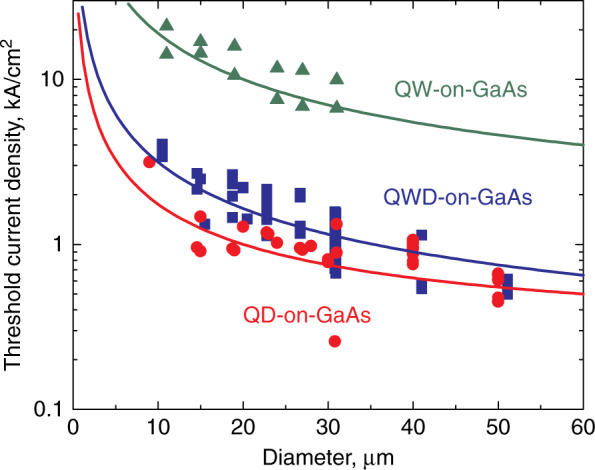
**Lasing threshold.** Threshold current density of microdisk lasers of different types against disk diameter for different active regions. Solid lines are binomial approximations

In Fig. 11, the threshold current densities $J_{{\mathrm{th}}}$ are summarized for microlasers of various types on GaAs substrates. The statistical scatter of the data is probably associated with some difference in the internal loss (varying from wafer to wafer) and in sidewall scattering (varying from one etching process to another). When both of these effects are at their minimum levels, low values of *J*_th_ are achieved, such as 257 A/cm^2^ that belongs to 31-µm QD-on-GaAs microdisk. In general, lower *J*_th_ among other counterparts are achieved in microlasers with these InAs/InGaAs Stranski-Krastanow QDs, where the majority of the experimental data can be described as:^55^ $J_{{\mathrm{th}}} \approx J_2 + 4j_1/D,$ with $J_2 \approx$ 250 A/cm^2^ and $j_1 \approx$ 0.38 A/cm. As *D* decreases from 50 to 15 µm, *J*_th_ rises rather slowly, from ~0.55 to ~1.2 kA/cm^2^ in average. We believe these features can be explained by the low transparency current density, relatively low optical loss, and suppressed non-radiative recombination in QD structures.

QWD-on-GaAs microdisks exhibit higher threshold current densities (on the order of kA/cm^2^) and stronger dependence on the diameter ($j_1 \approx$ 0.75 A/cm). We explain this by smaller localization energy (since their emission is shifted to shorter wavelengths compared to QD-on-GaAs) and, therefore, higher population of the waveguide with charge carriers, which can diffuse to the sidewalls. QW-on-GaAs microlasers^56,57^ are characterized by yet higher values of *J*_th_ ~ 10 kA/cm^2^; the $\propto 1/D$ term has an order of magnitude higher coefficient $j_1 \approx$ 4.5 A/cm. This is despite the fact that the localization energy there is quite large, since the emission wavelength is ~1.25 µm. The only reasonable explanation can be a stronger surface recombination, as the charge carriers in the active region have the ability to freely approach the edges of the disk.

### Microdisk thermal properties

**Fig. 12:**
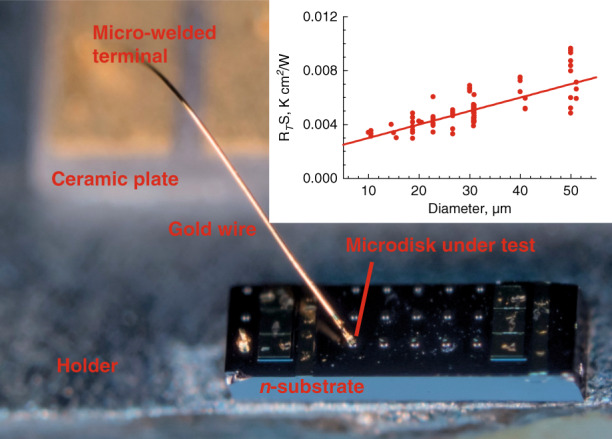
**Thermal resistance.** Main panel: chip with several microdisks of different sizes, mounted on holder, with wire welded to tested microdisk. Inset: thermal impedance by microdisk area as a function of diameter

Microdisk chips were mounted onto a copper holder, Fig. 12, and tested in continuous-wave regime without external cooling. In QD microdisk lasers with sufficiently large diameters the characteristic temperatures $T_0$ of ~90–100 K was found^58^, whereas in smaller microlasers, *J*_th_ increases sharply. It is attributed to a temperature increment caused by Joule heat. This effect also manifests itself through a redshift of the WGM lines, Fig. 9. Owing to a relatively low temperature coefficient (~0.08 nm/K), the redshift is small, but it increases with increasing electrical power consumed with a slope determined by the thermal resistance. The experimental data can be described, suggesting the one-dimensional heat flux through the mesa and the three-dimensional heat flow into the substrate with the mean thermal conductivities $\kappa _{{\mathrm{disk}}}$ and $\kappa _{{\mathrm{sub}}}$ of ~0.15 and 0.5 cm × W/K (solid line in inset to Fig. 12), which correlate with the thermal conductivity of Al_0.35_Ga_0.65_As and GaAs and, respectively^59^.

**Fig. 13:**
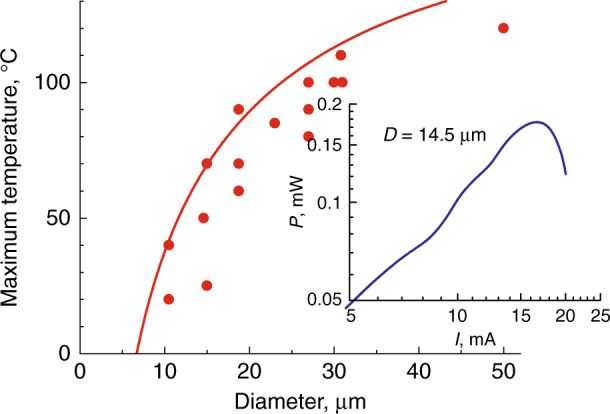
**CW operation of microdisk lasers.** Main panel: maximum temperature of CW operation vs microdisk diameter: experiment (QWD-on-GaAs) and simulation. Inset: light—current curve in CW regime; *P* - power at detector, *I* - injection current

Because of self-heating, there exists a minimum diameter, until which CW lasing can be realized; for a given microdisk size there is an upper limit of the operating temperature, Fig. 13. For example, *T*_max_ is 110 °C for 30-µm QWD-on-GaAs disk, but it drops to 40 °C for *D* = 10 µm. The maximum operation temperature we achieved with QD-on-GaAs microlasers is quite similar being 100 °C for a 30-µm disk. The model^60,61^, which takes into consideration the *J*_th_-*vs*-*D* relationship, satisfactorily describes the experimental data. Self-heating is also responsible for the thermal rollover behavior, inset to Fig. 13, revealed in the light-current curves of the microdisk lasers similar to the behavior reported for VCSELs^62–65^.

### High-speed performance

**Fig. 14:**
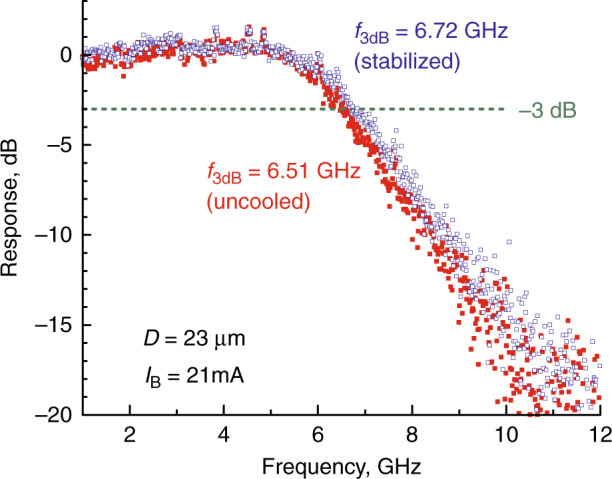
**Direct modulation of microdisk laser.** Small-signal frequency response of QWD-on-GaAs microdisk laser operating uncooled (filled symbols) or with temperature stabilization (open symbols) *f*_3dB_ - modulation bandwidth, *I*_B_ - bias current, *D* - disk diameter

It was revealed that QWD-on-GaAs microlasers are capable of providing high-output power emitted into free space. For example, in a 31 µm diameter QWD-on-GaAs microdisk we measured a maximum power of 18 mW, whereas a differential efficiency was ~31%^58^. This greatly simplifies (compared with other types of microlasers we studied) their characterization and, in particular, the measurements of high-speed modulation performance. Experiments on large signal modulation were performed using a 2^7^–1 pseudo random binary sequence; the microdisk emission was coupled to a lensed fiber and analyzed with a large bandwidth optical sampling module and a digital serial analyzer. Error-free data transmission was realized with a maximum speed of 10 Gb/s^66^. This correlates with the doubled values of −3 dB small-signal modulation bandwidth; $f_{3{\mathrm{dB}}}$ in the 6…7 GHz range was measured in QWD-on-GaAs microdisk lasers^67^. Our results are in agreement with the data reported for QD microrings^68,69^. Somewhat better performance was achieved for InP-based microlasers, which, however, require cooling as their temperature stability is poor (e.g., $f_{3{\mathrm{dB}}}$ of 14-μm InGaAsP/InP microdisk decreases from 20 to 12.4 GHz in the 14–40 °C interval^22^). In contrast, the absence of thermal stabilization of the QD microlaser leads, at a fixed bias, to a decrease in $f_{3{\mathrm{dB}}}$ by only 3%, Fig. 14.

**Fig. 15:**
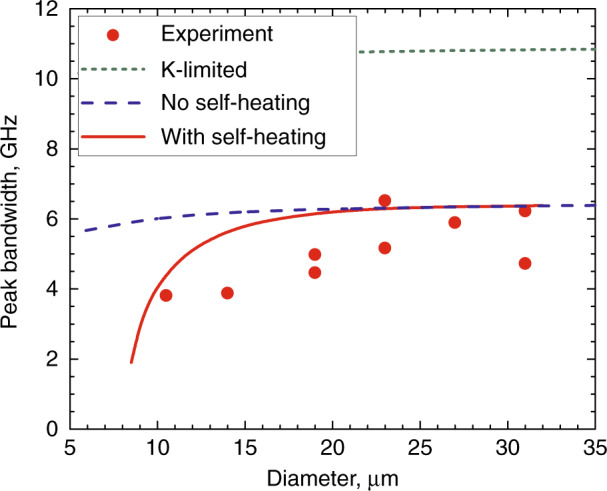
**Modulation bandwidths.** Peak modulation bandwidth depending on the diameter of the QWD-on-GaAs microdisk laser: symbols—experiment, lines—modeling for different scenarios

The dynamic performance of relatively large (>20 µm) injection microdisks was found to be limited by the capacitance-resistance time constant and capture times^70^. In smaller microdisks, the bandwidth is additionally affected by overheating ${\mathrm{{\Delta}}}T$, as $f_{3{\mathrm{dB}}} \propto \sqrt {I - I_{{\mathrm{th}}}({\mathrm{{\Delta}}}T)}$ ^71^, Fig. 15. Meanwhile, the K-factor limited modulation bandwidth, as extracted from the relationship between the damping rate and frequency of relaxation oscillations, is above 10 GHz^72^. Even higher peak bandwidth of 20 GHz was estimated from damped oscillations of the WGM intensity observed in optically pumped 6-µm-microdisk laser^73^.

**Fig. 16:**
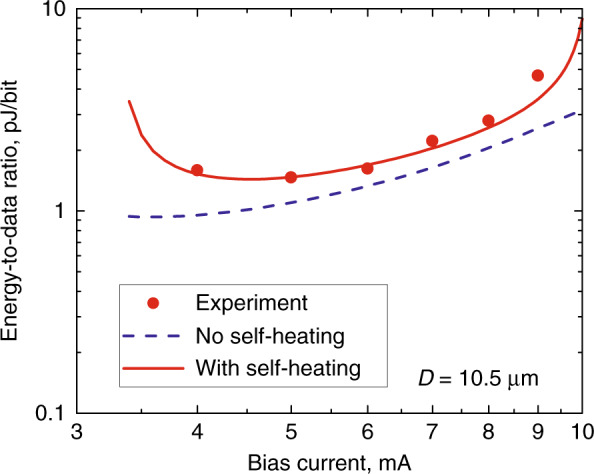
**Energy consumption per bit.** Energy-to-data ratio estimated from modulation characteristics against bias current: symbols—experiment (QWD-on-GaAs), lines—modeling with self-heating (solid line) or without (dashed line)

The energy-to-data ratio (EDR) is a figure-of-merit for a laser used for fata transmission^74^. EDR < 2 pJ/bit^55,75^ have been demonstrated for QWD-on-GaAs microdisk lasers. For QD microrings on silicon the estimate gives a minimum EDR of ~3.4 pJ/bit^68^. These values are lower ~7 pJ/bit previously reported for InP microlasers of comparable size^76^. The minimum EDR in the QWD-on-GaAs microdisk is achieved at a bias of about double *I*_th_ since a rapid decrease in *f*_3dB_ occurs at lower currents, whereas Joule heat dissipation increases at higher currents, Fig. 16. The EDR of 100 pJ/bit could be achieved in microdisk lasers with a diameter of ~4 μm if the problem of overheating of small microlasers were solved^55^.

### Microdisk lasers on silicon

**Fig. 17:**
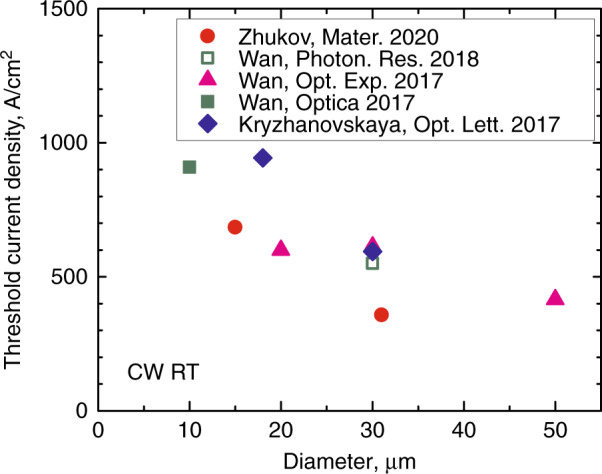
**Quantum dot microlasers on silicon.** Reported threshold current density of QD-on-Si microlasers

Success in synthesis of III–V materials on silicon would allow combining the capabilities of the CMOS technology and high-speed optical sources to create optoelectronic integrated systems for processing and transmitting information^77^. Despite the fact that the quality of III–V-on-Si materials is still inferior compared with those on GaAs, a reduced sensitivity of QDs to defects has made it possible to make a breakthrough in Si-based lasers^78,79^. The threshold current densities of QD broad-area laser on silicon (e.g., 62.5 A/cm^2 ^^78^) are not too far from the best values for those on GaAs. The threshold currents of QD microlasers, monolithically integrated with silicon, have also approached the values typical for GaAs, Fig. 17. In microring lasers on V-grooved Si, the lowest *J*_th_ increases from 0.42 to 0.61 kA/cm^2^ as an outer diameter is reduced from 50 to 30 µm^35^. We have recently report a lower value of 0.36 kA/cm^2^ in 31-µm-microdisk (QD-on-Si), operating in CW regime without thermal stabilization. Special mention should be made of microdisk/microring lasers hybridly integrated with Si or SOI substrates. For these purposes, heterostructures grown on InP substrates^80–82^ are usually used. It has been recently demonstrated that microlasers based on QD synthesized on GaAs substrates^36,83,84^ can be used as well.

In^83^, the temporal stability of the emitted power of the QD-on-Si microdisk laser was analyzed and the mean time to failure of 8 × 10^4^ h was estimated. This agrees with the estimated time to failure of 10^5^ h extracted for broad-area lasers made of similar epitaxial structures^79^. It should be noted that although it is significantly better than the values previously reported for QW lasers on Si substrates^85,86^, it is still an order of magnitude inferior to the lifetime of QD lasers on GaAs^87^.

## Conclusion

In conclusion, the use of self-organized QDs in macrolasers has led to a significant reduction of the laser’s threshold current density and drastic improvement of their temperature stability. QD lasers have demonstrated some unique properties such as the possibility to cover previously inaccessible spectral ranges, ultrawide gain spectra and lasing spectra, low-intensity noise of individual optical modes, etc. This, however, did not strongly change the market, which is still dominated by QW lasers. With the advent of microdisk/microring lasers, including microlasers on silicon, a niche appears for QDs, in which their specific properties are even more in demand, as they provide the very possibility of implementing such devices. The localization of charge carriers in spatially separated islands makes QD microlasers weakly sensitive both to defects arising from the synthesis on a non-native substrate and to those formed during the microcavity etching. As a result, the design of the device and its manufacturing technology can be significantly simplified without sacrificing its functionality. Investigations carried out in recent years have shown not only the fundamental possibility of creating such QD-based microlasers, but also demonstrated promising characteristics. For example, low-threshold microdisk lasers capable of operating at elevated temperatures have been reported; robustness against deep etching was revealed; monolithic or hybrid integration with silicon was implemented; some preliminary, but promising results on high-speed operation, data transmission, and reliability have been achieved. All this supports our expectations regarding the possible use of these lasers for optical interconnects and on-chip optical sources.

Our experimental findings confirm that 3D localization of charge carriers in the active region is a key factor for high-performance microlasers. For example, it was demonstrated that acceptably low-threshold current densities can be achieved in microlasers of small diameter with either InAs/InGaAs Stranski-Krastanow QDs (QD-on-GaAs) or InGaAs dense quantum dots (QWD-on-GaAs), whereas quantum well-based microlaser (QW-on-GaAs) suffer from significant surface recombination. When comparing microlasers based on different types of quantum dots (QD-on-GaAs and QWD-on-GaAs), we found that deeper localization energy contributes to better threshold characteristics, which can be explained by more complete suppression of charge carrier transport along the GaAs matrix waveguide. Meanwhile, the higher density of islands in QWD-on-GaAs structures is possibly the reason for their higher external efficiency compared to QD-on-GaAs, which favors their use in various applications, including optical data transmission.

The recent studies have also revealed a number of issues to be addressed. First of all, this concerns a further reduction in the power consumption and heat dissipation, which should make it possible to realize the high-speed operation of microlasers with diameters of several micrometers rather than several tens of micrometers. Another serious challenge is the suppression of parasites that reduce the modulation speed. One can also hope for the development of various schemes for microlaser integration with functional substrates and photonic elements. In particular, a possible way to integrate a III–V-based emitter with silicon is to transfer a prefabricated QD-based device or an array of devices onto a silicon surface with the use of a soft intermediate paste capable of compensating for the difference in thermal expansion coefficients. The very first steps in this direction have been made recently.

## Data Availability

The data that support the conclusions within this paper and the other findings of this study are available from the corresponding authors upon reasonable request.
